# Comparison of awareness about precautions for needle stick injuries: a survey among health care workers at a tertiary care center in Pakistan

**DOI:** 10.1186/s13037-016-0108-7

**Published:** 2016-09-07

**Authors:** Abdul Rafay Qazi, Furqan Ali Siddiqui, Salman Faridi, Urooj Nadeem, Nida Iqbal Umer, Zainab Saeed Mohsini, Muhammad Muzzammil Edhi, Mehmood Khan

**Affiliations:** 1 Department of General Surgery, Liaquat National Hospital and Medical College, Karachi, Pakistan; 2 Liaquat National Hospital and Medical College, Karachi, Pakistan; 3 Dhaka Medical College, Karachi, Pakistan

**Keywords:** Needle stick injuries, Health care workers, Awareness, Laboratory technicians, Nursing staff, Doctors

## Abstract

**Background:**

Needle stick injuries (NSIs) have the potential of causing Hepatitis B and Hepatitis C, which is constantly adding to the burden of chronic liver disease in our country. It poses a risk to Health Care Workers (HCWs) and the patients they deal with. In order to limit the spread of these viruses, it is imperative that these HCWs be fully equipped with knowledge regarding prevention of NSIs and dealing with one, regardless of their designation. We therefore aimed to assess and compare the level of awareness about precautions for needle stick injuries amongst all those greatest at risk.

**Methods:**

This was a cross- sectional study carried out at Liaquat National Hospital, Karachi, Pakistan. A 23 itemed self-administered questionnaire was given to hospital staff including doctors, lab technicians and nurses via convenience sampling, in various departments. Data was analyzed via SPSS 18 software and a *p*-value of <0.05 was considered significant.

**Results:**

A total of 198 responses were taken for this study, out of which 70 (35.4 %) were doctors, 70 (35.4 %) nursing staff and 58 (29.3 %) laboratory technicians. Of all HCWs, 101 (51 %) knew that the standard method of discarding needles is without recapping. 159 (80.3 %) were still recapping needles. 180 (90.9 %) HCWs were vaccinated against Hepatitis B. 36 (18.2 %) were aware that blood should be allowed to flow after an NSI and site of prick should be washed with an antiseptic.

**Conclusion:**

The awareness was found to be very low amongst all HCWs. It should therefore be made compulsory for all HCWs to attend proper preparatory classes by the infection control department at the time of employment in order to improve the level of awareness and ensure safe practices.

**Electronic supplementary material:**

The online version of this article (doi:10.1186/s13037-016-0108-7) contains supplementary material, which is available to authorized users.

## Background

Needle stick and sharps injuries (NSIs) have been recognized as one of the many Occupational hazards among health care workers (HCWs) [1]. Needlestick injury is defined as any percutaneous injury with sharp equipment used in the delivery of medical care. Such equipment may include hollow-bore needles, suture needles, scalpels, IV equipment, etc. [2].

The pathogens of greatest concern that may be transmitted by NSI are hepatitis B (HBV), hepatitis C (HCV), and human immunodeficiency virus (HIV). While other blood borne pathogens, including Hepatitis G, Herpes Simplex 1, Group A Streptococcus and Human Parvovirus B19 may also be transmitted by NSI, they are less common [2–9].

An estimated 600,000 to 800,000 needle stick and other percutaneous injuries are reported annually among U.S. HCWs [10]. It is estimated that 100,000 needle stick injuries occur annually in UK alone and 500,000 annually in Germany [11, 12].

Hepatitis B virus (HBV) is a DNA virus and one of many unrelated viruses that cause viral hepatitis and can lead to liver cirrhosis and hepatocellular carcinoma. More than three-quarters of its infections occur in Asia, Middle East and Africa. According to a WHO estimate, two billion people in the world have serological evidence of prior HBV infection (WHO, 2000). Of the world’s carriers of HBV, 75 % are from Asia.

The incidence and prevalence of chronic liver disease due to HBV and HCV is gradually increasing in Pakistan. An increasing number of these patients are brought to tertiary care hospitals for diagnosis and management. This puts HCWs and the patients they deal with, at an ever growing risk of exposure to these blood borne pathogens. Even though there are many sources of spread of these blood borne pathogens, sharps injuries remain a major source of HCV infection among HCWs, accounting for almost 40 % of HCV infections. Contaminated sharps were estimated to cause 66,000 HBV infection annually, associated with 261 deaths [17].

According to a survey conducted by Pakistan Medical Research Council from July 2007 to May 2008, the burden of hepatitis B in Pakistan is 2.5 % and that of hepatitis C is 5 % in general population.

WHO reports in the World Health Report 2002, that of the 35 million health-care workers, 2 million experience percutaneous exposure to infectious diseases each year. It further notes that 37.6 % of Hepatitis B, 39 % of Hepatitis C and 4.4 % of HIV/AIDS in Health-Care Workers around the world are due to needle stick injuries [10].

Certain groups of individuals are at greater risk than others because of the nature of their work especially the health care workers because they handle sharp devices or equipment’s such as scalpels, sutures, hypodermic needles, blood collection devices or phlebotomy devices. The most common sharps injury is caused by needles [13]. Numerous studies have found nurses to be the commonest group of HCWs experiencing needle stick injuries [13].

In the developing nations worldwide the incidence of these blood borne diseases are higher as compared to the developed nations because of illiteracy, poverty, malpractice of sharp objects, deficient training, ignorance, lack of resources for disposing them.

According to World Health Organisation (WHO) regional classification, Pakistan comes in Eastern Mediterranean Region D (EMR D). Unfortunately this region has the highest rate of needle stick injuries as compared to the entire world [14, 15].

The objective of this study was to compare awareness and compliance about precaution of needle stick injuries amongst doctors, nursing staff and technicians at Liaquat National Hospital. Comparison was done in order to assess whether all the three professions had identical level of awareness as they are equally prone to acquire accidental needlestick injuries.

## Methods

This was a cross sectional study conducted by the department of emergency medicine at Liaquat National Hospital, Karachi, Pakistan, which is one of the largest private, tertiary health care facility and teaching hospital. The study was approved by Liaquat National Hospital and medical college, Karachi, Pakistan ethical committee. It was done to compare the awareness and compliance about precautions for needlestick injuries amongst doctors, nursing staff, and technicians working in different departments of LNH that perform invasive and non-invasive procedures.

The data was collected by means of a structured questionnaire, from June to August 2012. Informed consent was taken and assurance given that all information would be confidential and would not be used for any purpose other than research.

The questionnaire was based on information related to knowledge about standard practices at workplaces, universal methods of disposal of sharps, spreads of diseases through NSIs and its causes its frequency, and ways of dealing with NSIs, once experienced, were assessed. Name and Gender was not taken in account for acquiring NSIs (Additional file 1).

We counted the correct number of responses on the questionnaires and used the percentage of correct responses to determine the level of awareness of HCWs.

The data was analyzed in SPSS 18 for frequency and percentages. Chi square test was used as a test of significance and p-value was fixed at 0.05 to be statistically significant.

## Results

We received 198 responses for this study of which, out of which 70 (35.4 %) were doctors, 70 (35.4 %) were nursing staff and 58 (29.3 %) were laboratory technicians.

It was found that the majority of people 117 (59.9 %) were somewhat aware, as shown in the Table 1. Doctors and nursing staff were found to have an equal level of awareness. Chi square test was done to evaluate if significance existed between designation and level of awareness but no significance was discovered. *P*-value was 0.298.

**Table 1:** Level of awareness amongst the 3 groups of health care workers

	Not aware (10–29)	Somewhat aware (30–49)	Aware (50 and above)
Doctors	9(22.5 %)	46(39.3 %)	15(36.6 %)
Nursing staff	19(47.5 %)	36(30.8 %)	15(36.6 %)
Technicians	12(30.0 %)	35(29.9 %)	11(26.8 %)
Total	40(20.2 %)	117(59.1 %)	41(20.7 %)

The level of awareness was also correlated to how well it caused them to adopt precautionary measures. It was found that 58 (100 %) laboratory technicians, 65 (91.4 %) doctors and 65 (92.9 %) nurses used gloves for standard procedures as shown in Table 2. But it was found that even though 101 (51 %) HCWs knew that the standard method of discarding needles is without recapping as shown in Table 3, only 39 (19.7 %) disposed needles without recapping. Of these, majority were the nursing staff 22 (31.4 %), lab technicians 10 (17.2 %) and only doctors 7 (10 %). 159 (80.3 %) HCWs were practicing recapping needles, majority of which were doctors, 63 (39.6 %) and 42 (30.2 %) were lab technicians and nursing staff as shown in Table 4.

**Table 2:** Adopting precautionary measures i.e. using gloves for standard procedures

	Yes	No	Total
Doctors	65(92.9 %)	05(7.1 %)	70
Nursing staff	65(92.9 %)	05(7.1 %)	70
Technicians	58(100 %)	00(0 %)	58
Total HCWs	188(94.9 %)	10(5.1 %)	198

**Table 3:** Knowhow of standard method of discarding needles i.e. without recapping

Yes	No	Total
101(51 %)	97(49 %)	198

**Table 4:** Practicing method of discarding needles

	Without recapping	Recapping	Total
Doctors	07 (17.9 %)	63 (39.6 %)	70
Nursing staff	22 (56.4 %)	48 (30.2 %)	70
Technicians	10 (25.7 %)	48 (30.2 %)	58
Total	39	159	198

The prevalence of NSIs was 99 (50 %) as shown in Table 5 and out of these, 31 (31.3 %) had experienced an NSI while recapping. Only 24 (24.2 %) people who experienced an NSI were aware enough to take post exposure prophylaxis, a greater number of which were the lab technicians 11 (45.8 %), 7 (29.2 %) nursing staff and 6 (25 %) doctors as shown in Table 6.

**Table 5:** Frequency of NSIs

Yes	No	Total
99(50 %)	99(50 %)	198

**Table 6:** Post Exposure Prophylaxis(PEP)

	PEP taken	PEP not taken	Total
Doctors	06 (8.6 %)	64 (91.4 %)	70
Nursing staff	07 (10 %)	63 (90 %)	70
Technicians	11 (19 %)	37 (81 %)	58
	24	164	198

It was further evaluated that 177 (89.4 %) HCWs were aware that Hepatitis B spreads through needle stick injuries 64 were doctors, 57 nurses and 56 lab technicians as shown in Table 7. 180 (90.9 %) HCWs had received at least 1 dose of vaccination against Hepatitis B. The majority, that is 13 (6.6 %), that did not consider Hepatitis B to spread from needle stick injuries was the nursing staff, compared to 6 (3 %) doctors and 2 (1 %) lab technicians as shown in Table 7. About 27 (15 %) of those who had not completed the vaccination course of 3 doses, majority were also the nursing staff 12 (6.7 %). 153 (77.3 %) HCWs had completed their vaccination course of 3 doses.

**Table 7:** Awareness of Hep B spread through NSIs

	Aware	Not aware	Total
Doctors	64(91.4 %)	06(8.6 %)	70
Nursing staff	57(81.4 %)	13(18.6 %)	70
Technicians	56(96.5 %)	02(3.5 %)	58
Overall	177(89.4 %)	21(10.6 %)	198

Around 15 (7.6 %) did not know that Hepatitis C spreads through NSIs. Of these, 10 (66.7 %) were nursing staff and 5 (33.3 %) were doctors as shown in Table 8. 38 (19.4 %) HCWs had the perception that the vaccine was against Hepatitis C, of which the majority were the lab technicians 17 (44.7 %), nursing staff 16 (42.1 %) and doctors 5 (13.2 %). 27 (13.6 %) were unaware that HIV spreads through NSIs. Of these, 19 (70.4 %) were from the nursing staff, 6 (22.2 %) lab technicians and 2 (7.4 %) doctors as shown in Table 9.

**Table 8:** Awareness of Hep C spread through NSIs

	Aware	Not aware	Total
Doctors	65(92.8 %)	05(7.2 %)	70
Nursing staff	60(85.7 %)	10(14.3 %)	70
Technicians	58(100 %)	00	58
Overall	183(92.4 %)	15(7.6 %)	198

**Table 9:** Awareness of HIV spread through NSIs

	Aware	Not aware	Total
Doctors	68(97.1 %)	02(2.9 %)	70
Nursing staff	51(72.8 %)	19(27.2 %)	70
Technicians	64(91.4 %)	06(8.6 %)	58
Overall	171(86.4 %)	27(13.6 %)	198

Of those who had completed their vaccination course, only 64 (41.8 %) ever received a booster dose, out of which 28 (43.8 %) were nurses, 19 (29.7 %) lab technicians and 17 (26.6 %) doctors as shown in Table 10.

**Table 10:** Receiving booster dose

	Yes	No	Total
Doctors	17(24.3 %)	53(75.7 %)	70
Nursing staff	28(40 %)	42(60 %)	70
Technicians	19(27.1 %)	39(72.9 %)	58
Total HCWs	64(32.3 %)	134(67.7 %)	198

Those who had not been vaccinated were further questioned about the reason for not getting vaccinated and it was noted that 13 (72.2 %) were aware but not bothered to receive vaccination as shown in Table 11.

**Table 11:** Reason of not getting vaccinated

	Aware but not bothered	Not aware	Financial issue
Doctors	05	00	01
Nursing staff	04	00	02
Technicians	04	01	01
Overall	13	01	04

HCWs were also questioned about what their immediate response to NSIs should be, according to Universal Precaution guidelines. It was found that 93 (47 %) were aware that blood should be allowed to flow after an NSI and 31 (15 %) that site of prick should be washed with running water. Most HCWs, that is 119 (60.1 %), said that the site of pricked should be washed with an antiseptic. 108 (54.5 %) knew that viral serology of both patient and person receiving injury should be tested. Of these, the majority was the nursing staff 38 (35.2 %) and 35 (32.4 %) doctors and lab technicians each as shown in Table 12.

**Table 12:** Awareness of Viral Serology

	Patient	Person	Both	No one
Doctors	22	11	35	02
Nursing staff	09	17	38	06
Technicians	12	10	35	01
Overall	43	38	108	09

## Discussion

Our study showed that, 21 (10.6 %) HCWs were still unaware that Hepatitis B spreads through needle stick injuries, 15 (7.6 %) about Hepatitis C and 27 (13.6 %) about HIV, similar to a study conducted at the Holy Family Hospital in Rawalpindi hospital in 2008, which showed that 40 (13.3 %) HCWs were unaware of the fact that Hepatitis B can be transmitted by NSI and 30 (10 %), about Hepatitis C [18]. The vaccine against HBV infection has been available since 1982. Hepatitis B vaccine is 95 % effective in preventing HBV infection and its chronic consequences, and it is the first vaccine against a major human cancer. However, vaccination rates in Elizabeth et al. study have been found to be low among health care providers although due to their level of exposure were supposed to have higher vaccine coverage rates [16].

Even though a good number of respondents, 158 (80.6 %), were aware about the availability of HBV vaccine in our study, only 153 (77.3 %) had completed their vaccination course, which shows data similar to a recent study in Kuwait where 81.5 % were aware of HBV vaccine, 65.9 % were aware about the number of doses of vaccination required for complete protection and 84.0 % had completed the vaccination doses. Various studies show that there are many potential reasons for not being vaccinated such as busy schedules, being very careful and cannot be infected from patient, lack of knowledge about severity and vaccine efficacy, perception of low risk status, the bother of a sore arm [16].

Similarly our study also showed that awareness was not the major issue for not receiving vaccination, rather, of the (9.1 %) that did not receive vaccination, 13 (72 %) HCWs were aware but not bothered.

Our study also showed that, awareness regarding universal precautions was higher amongst medical doctors, 36.6 %, and nurses 36.6 %, as compared to lab technicians 26.8 %. Our results differ slightly from that of K. Vaz, D. McGrowder, et al. in which nurses were 90 % knowledgeable, followed by medical doctors, 88 % and medical technologists, 70 % [14]. The reason for this could be attributed to the fact that curriculum in medical colleges and nursing school was adequate enough to make them well aware regarding NSI’s while Lab technicians lacked as they relied mainly on infection control classes, and hence had a lower level of awareness, comparatively.

In the study by K. Vaz, D. McGrowder, et al. 59.3 % of HCWs always recapped the needle after use whilst in our study 153 (80.3 %) HCWs were practicing recapping needles, because of this 31 (31.3 %) HCWS had experienced an NSI while recapping [17]. Out of 153 (80.3 %) HCWs practicing recapping, the least number was that of the nursing staff 42 (30.2 %) and lab technicians as compared to a higher number of doctors 39.6 %. Similar results were reported in research conducted in Ghurki Trust Teaching Hospital, Lahore, where 32.1 % NSIs were experienced by nursing staff at the time of recapping the syringe [18].

These practices are prevailing all over the world even though according to the USA OSHA’s blood-borne pathogen standards (1996), in order to reduce the risk of transmission of blood-borne pathogens recapping a needle is prohibited. But a greater majority of NSIs in our study was due to drawing blood, 42 (42.4 %) which was equivalent to a study carried out at Aga Khan Hospital, Pakistan which reported that more than half of the injuries, 52.8 %, occurred while drawing the blood samples or injecting the medicine [19, 20]. In our study 187 (94.4 %) HCWs used gloves while performing standard procedures, of which the technicians were the majority 58 (100 %), whereas in the United States, a study conducted in two privately owned community hospitals in Minneapolis reported that gloves were observed to be used when appropriate only 67.2 % of the time [21].

About 40-70 % cases of needle stick injuries remain unreported in developing countries, similarly 11 (15.7 %) doctors in our study thought that a needlestick injury need not be reported and another research showed that doctors especially surgeons were least number in reporting NSIs, which could be most likely because of self-assessment of low risk and likelihood of self-care for injuries [15]. It is important to note, therefore, that due to insufficient information retention, knowledge and adherence to taught practice may still be deficient in spite of proper training and education [22–28].

## Conclusion

The awareness was found to be very low amongst all HCWs. It should therefore be made compulsory for all HCWs to attend proper preparatory classes by the infection control department at the time of employment in order to improve the level of awareness and ensure safe practices.

We recommend a good reporting system should be developed in order to identify the areas of greater risk so more focus can be given to them in order to prevent the occurrence as well as for providing proper and immediate care to the worker receiving injury.

## Additional file

Additional file 1: Serial No. (DOCX 15 kb)
