# Supplementary material for: Comparison of awareness about precautions for needle stick injuries: a survey among health care workers at a tertiary care center in Pakistan
Source: Patient Saf Surg. 2016 Sep 7;10(1):19. doi: 10.1186/s13037-016-0108-7 (PMC5015332; doi:10.1186/s13037-016-0108-7)
Supplement: Additional file 1: — Serial No. (DOCX 15 kb) [file 13037_2016_108_MOESM1_ESM.docx]

**Performa: Comparison of awareness about precautions for needle stick injuries: a survey among health care workers at a tertiary care center in Pakistan.**

Serial No:

Name (Optional):

Designation:

Department:

1. Have you heard about Needle stick injury? A) Yes b) no

2. From which source

a. Internet

b. Class

c. Book

d. Colleague

e. Journal

f. Media

4. Do you use gloves for standard procedures as a precautionary measure?

a. yes

b.no

5. Do you recap needle after use to dispose it?

a. yes

b.no

6. Do you know standard method of discarding needle is?

a. Without recapping

b. Recapping with one hand

c. Recapping with both hands

7. Do you know NSI can transmit?

Hepatitis B Yes No

Hepatitis C Yes No

HIV Yes No

9. Perception of vaccine against Hep B or Hep C?

10. Have you received at least 1 dose of vaccination against Hep B or Hep c a) yes b) no

13. How many vaccination doses you have received against Hep B?

a) 1 b) 2 c) 3

15. Have you ever sustained needle stick injury?

a) Yes b) no

16. How often you have sustain needle stick injuries?

A. once

B. twice

C. more than twice

17. In which department you sustained NSI?

a. ER

b. OT

c. Ward

d. ICU

18. Can you recall when you sustained NSI?

a. While injecting IM

b. Drawing blood for testing

c. While doing cannulation

d. While recapping syringe

e. While suturing

F. During surgery

G. From your HCW colleague

19. Are you aware of taking post exposure prophylaxis after sustaining NSI?

A) Yes b) no

20. After sustaining NSI what was your immediate response?

a. Allowed blood to flow out

b. Pressed the site of injury

c. Washed with antiseptic solution at site of injury

d. Immediately washed with running water.

21. After sustaining NSI to whom you notified?

a. Occupational Health Department

b. Infection control department

C. To your own department

d. None

E. To your supervisor

22. After NSI, who should be tested for viral serology?

a. Patient

b. Person sustaining injury

c. Both

23. How recently you have been exposed to NSI?

a. Recently

b. 3months back

c. 1year back
